# Supplementary material for: A game theoretic approach identifies conditions that foster vaccine-rich to vaccine-poor country donation of surplus vaccines
Source: Commun Med (Lond). 2022 Aug 23;2:107. doi: 10.1038/s43856-022-00173-w (PMC9395896; doi:10.1038/s43856-022-00173-w)
Supplement: Supplementary file 1 — Description of Additional Supplementary Files [file 43856_2022_173_MOESM1_ESM.pdf]

Description of Additional Supplementary Files

File Name: Supplementary Data 1

Description: Raw data for Figure 1

File Name: Supplementary Data 2

Description: Raw data for Figures 2-5
