# Supplementary material for: A game theoretic approach identifies conditions that foster vaccine-rich to vaccine-poor country donation of surplus vaccines
Source: Commun Med (Lond). 2022 Aug 23;2:107. doi: 10.1038/s43856-022-00173-w (PMC9395896; doi:10.1038/s43856-022-00173-w)
Supplement: Supplementary file 2 — Supplementary Material [file 43856_2022_173_MOESM2_ESM.pdf]

# Supplementary information

## A game theoretic approach identifies conditions that foster vaccine-rich to vaccine-poor country donation of surplus vaccines

Adam Lampert[1], Raanan Sulitzeanu-Kenan[2], Pieter Vanhuysse[3], Markus Tepe[4]

1. Institute of Environmental Sciences, Robert H. Smith Faculty of Agriculture, Food and Environment, The Hebrew University of Jerusalem, Rehovot, Israel; 2. Federmann School of Public Policy, The Hebrew University, Jerusalem, Israel; 3. Department of Political Science and Danish Institute for Advanced Study, University of Southern Denmark, Odense, Denmark; 4. Institute of Social Sciences, University of Oldenburg, Oldenburg, Germany.

## Supplementary Note 1: Estimating the parameter values in the context of the COVID-19 pandemic

### Estimating $v_{\mathbf{max}}$

Given a world population of 7.9 billion (as of Oct. 2021); the share of vaccinated globally is .4216 (fully vaccinated) and .1139 (partially vaccinated). We estimate the global proportion of the vaccinated population at .4786 (.4216+.1139/2) (https://ourworldindata.org/covid-vaccinations. Data extracted on November 24, 2021). Thus, the number of unvaccinated is estimated at 4.119 billion (.52145×7.9).

Based on this estimate and on the number of ordered vaccine doses around the world (missing data for Russia and Turkmenistan), we can provide some estimates for $v_{\max}$ (https://www.economist.com/graphic-detail/tracking-coronavirus-across-the-world, data extracted on October 26, 2021). This measure links our estimates to existing production capacity, rather than merely the willingness of countries to invest in vaccinations. Since these data exclude stocking in the form of a dedicated budget, our analysis here yields a conservative estimate for both $v_{\max}$.

If we order countries by the size of their surplus doses stock, the first two political entities – the European Union and the United States – offer $v_{\max} = .53$; and the first ten provide $v_{\max} = .85$. Noted that the sizes of surplus doses vary between countries, while our theoretical model assumes that vaccine-rich countries are equal.

### Estimating $\alpha$

We assume that a country is only required to re-vaccinate its population in the advent of a new outbreak due to a VOC. One way to approximate $\alpha$ is to estimate the time it takes to administer the vaccine to a sizable proportion of the population (50%), vis-à-vis the length of an outbreak wave (absent a vaccine). Based on the duration of the second outbreak of COVID-19 in 12

countries (COVID-19 Data Explorer), a typical outbreak lasts 5.9 months (SD=1.83), but we assume its costs (health implications and economic disruption) linger further up to 10 months. If a country has vaccines available to provide a booster shot, it will incur the cost of the outbreaks until it manages to re-vaccinate a sizable proportion of its population. The average time it took the five vaccine-rich countries (Supplementary Table 1) to reach this level of vaccination during 2021 is 6.19 months. However, in some of these countries, delays in vaccinations were due to vaccine shortages (see: New York Times, January 27, 2021). Thus, assuming no shortages and given that a booster shot requires just one dose, this duration may be only 3-4 months, yielding a plausible range of $0.3 < \alpha < 0.6$.

**Supplementary Table 1:** Durations of public vaccination (Source: https://ourworldindata.org/covid-vaccinations. Data extracted on November 24, 2021).

| Country | Time to 50% fully vaccinate (in months) |
|---|---|
| Canada | 6.72 (12.12.2020-7.19.2021) |
| France | 6.69 (1.16.2021-8.7.2021) |
| Germany | 7.00 (12.27.2020-7.27.2021) |
| Israel | 3.10 [12.19.2020-3.24.2021] |
| United Kingdom | 5.90 (1.10.2021-7.7.2021) |
| United States | 7.77 (12.13.2020-8.6.2021) |
| Average | 6.20 [95% CI: 4.48-7.9] |

### Estimating $\lambda$

As noted in the main text, the utility of stocking surplus vaccine doses is associated with the likelihood of VOCs. The rate of VOC emergence is, to the best of our knowledge, unknown. A tentative assessment of the two-year experience with known COVID-19 VOCs suggests that, while there have been infections with VOCs among vaccinated, vaccine efficacy in reducing the severity of disease was retained for the first four VOCs[1]. However, at present, it is still unknown whether the fifth VOC reported recently by the WHO (B.1.1.529, Omicron) threatens vaccine effectiveness[2]. Given this evidence, we cautiously maintain that a plausible range for the rate of VOCs per year is 0.5-1.5. To the extent that more accurate estimates are available, either in the context of the current pandemic or the next, we considered an even wider range of $0 \leq \lambda \leq 2$.

**Supplementary References**

1. Tregoning, J. S., Flight, K. E., Higham, S. L., Wang, Z. & Pierce, B. F. Progress of the COVID-19 vaccine effort: viruses, vaccines and variants versus efficacy, effectiveness and escape. *Nat. Rev. Immunol.* **21**, 626–636 (2021).

2. Callaway, E. *Heavily mutated Omicron variant puts scientists on alert. Nature.* ***600**, 21* (2021).
